# Role of Synthetic Parameters on the Structural and Optical Properties of N,Sn-Copromoted Nanostructured TiO_2_: A Combined Ti K-Edge and Sn L_2,3_-Edges X-ray Absorption Investigation

**DOI:** 10.3390/nano10061224

**Published:** 2020-06-23

**Authors:** Martina Fracchia, Paolo Ghigna, Alessandro Minguzzi, Alberto Vertova, Francesca Turco, Giuseppina Cerrato, Daniela Meroni

**Affiliations:** 1 Department of Chemistry, Università degli Studi di Pavia, via Taramelli 12, 27100 Pavia, Italy; paolo.ghigna@unipv.it; 2 Consorzio Interuniversitario per la Scienza e Tecnologia dei Materiali (INSTM), via Giusti 9, 50121 Florence, Italy; alessandro.minguzzi@unimi.it (A.M.); alberto.vertova@unimi.it (A.V.); 3 Department of Chemistry, Università degli Studi di Milano, via Golgi 19, 20133 Milan, Italy; 4 Department of Chemistry and NIS, Inter-Departmental Center, Università degli Studi di Torino, Via P. Giuria 7, 10125 Torino, Italy; francesca.turco@unito.it (F.T.); giuseppina.cerrato@unito.it (G.C.)

**Keywords:** X-ray absorption spectroscopy, TiO_2_ nanoparticles, photocatalysis

## Abstract

Sn-modification of TiO_2_ photocatalysts has been recently proposed as a suitable strategy to improve pollutant degradation as well as hydrogen production. In particular, visible light activity could be promoted by doping with Sn^2+^ species, which are, however, thermally unstable. Co-promotion with N and Sn has been shown to lead to synergistic effects in terms of visible light activity, but the underlying mechanism has, so far, been poorly understood due to the system complexity. Here, the structural, optical, and electronic properties of N,Sn-copromoted, nanostructured TiO_2_ from sol-gel synthesis were investigated: the Sn/Ti molar content was varied in the 0–20% range and different post-treatments (calcination and low temperature hydrothermal treatment) were adopted in order to promote the sample crystallinity. Depending on the adopted post-treatment, the optical properties present notable differences, which supports a combined role of Sn dopants and N-induced defects in visible light absorption. X-ray absorption spectroscopy at the Ti K-edge and Sn L_2,3_-edges shed light onto the electronic properties and structure of both Ti and Sn species, evidencing a marked difference at the Sn L_2,3_-edges between the samples with 20% and 5% Sn/Ti ratio, showing, in the latter case, the presence of tin in a partially reduced state.

## 1. Introduction

The promotion of TiO_2_ photocatalysts with Sn species, either by doping with Sn ions or by composite formation with SnO*_x_*, has been successfully applied to numerous fields, such as the photocatalytic degradation of water [1,2] and air pollutants [3,4,5], photovoltaics [6], and hydrogen production [7,8]. Because one of the main disadvantages of TiO_2_ photocatalysts is their limited activity in the visible region, due to the large band gap (≥3.0 eV) [9], doping with Sn^2+^ species has raised interest as a strategy for promoting TiO_2_ visible light activity [1,2,3,5,8]. Visible light absorption of TiO_2_ doped with Sn^2+^ has been related to a band gap narrowing caused by a shift in the position of the valence band, consisting of Sn 5s and O 2p orbitals [3]. Martinez-Oviedo et al., on the contrary, reported an extensive formation of Ti^3+^ species in Sn^2+^-doped TiO_2_, leading to a marked blue color [5].

The reported syntheses of Sn^2+^-doped TiO_2_ use SnCl_2_ as a starting material and require reducing conditions [5,8], leading to materials with limited thermal stability [1,10,11]. As a matter of fact, thermal treatments in non-reducing atmosphere result in the conversion of Sn^2+^ in Sn^4+^, leading to the loss of visible-light absorption.

Doping with Sn^4+^ has been reported to be beneficial to TiO_2_ activity, but only under UV irradiation [4]. Indeed, DFT calculations [11,12] have shown no effect or a blue shift in the absorption edge of anatase and rutile TiO_2_ when Sn^4+^ substitutional doping occurs; the latter has been reported to be the most energetically favored situation at low dopant content [11,12,13]. Conversely, the surface segregation of SnO*_x_* clusters has been often observed at higher Sn content [4,13,14]. The formation of heterojunctions with SnO_2_, while beneficial for the charge separation of photogenerated carriers [15,16], does not promote visible light activity of the resulting composite due to the large band gap of SnO_2_ [17]. However, the doping of SnO_2_ by Sn^2+^ has been recently reported to induce visible light activity in the semiconductor. Additionally, in this case, reducing conditions are required during the synthesis, leading to yellowish powders [18,19].

As an alternative strategy to induce visible light activity in TiO_2_ materials, copromotion with N and Sn^4+^ has been recently proposed: this approach leads to synergistic effects between the two dopants in terms of visible light absorption [6,20], but the underlying mechanism has, so far, been poorly understood.

Most of the reports on Sn-doped TiO_2_ investigate the oxide structural features and the dopant oxidation state by X-ray diffraction (XRD) and X-ray photoelectron spectroscopy (XPS), respectively, with only few reports using other techniques, such as Mössbauer spectroscopy [6,10,11] and X-ray absorption (XAS) techniques, like extended X-ray absorption fine structure (EXAFS) [11,21] and X-ray absorption near edge structure (XANES) [3,21,22]. To the authors’ best knowledge, the latter are exclusively limited to K-edge spectra of Sn. X-ray absorption spectroscopy is expected to give additional and complementary information with respect to that achievable by XRD or XPS. XPS is, in fact, a surface technique, providing more information regarding the Sn species segregated at the particle surface. It is mandatory to probe the oxidation state and chemical surrounding of tin in the bulk in order to find correlations between the electronic structure and bulk physical properties, such as light absorption. Moreover, a precise identification of phases (regarding both Ti and Sn) is difficult in the case of XPS analysis, where peak interpretation is often misleading, as well as of XRD measurements, due to the reflections broadening caused by the sample nanocrystalline nature. On the contrary, X-ray absorption spectroscopy is a bulk technique and it is element selective, hence enabling us to gather electronic and structural information selectively on both tin and titanium. In this respect, it should be noted that the diverse TiO_2_ polymorphs yield different and easily distinguishable XANES spectra at the Ti K-edge [23,24], while SnO_2_ and SnO show different response at the Sn L_2,3_-edges, thus allowing us to detect modifications in the tin valence state [25,26,27]; in fact, 5*s* states can be directly probed at the Sn L_2,3_-edges.

Some of us have recently reported about N,Sn-copromoted, nanostructured TiO_2_ from sol-gel synthesis with enhanced photocatalytic properties towards the degradation of tetracycline, an emerging water pollutant [20,28]. Interestingly, different light absorption could be attained, depending on the type of applied thermal post-treatment (calcination or low temperature hydrothermal treatment), with ensuing different photocatalytic activity of the materials in the UV-vis region. Here, we report a combination of X-ray absorption and UV-visible light absorption experiments, which aimed to shed light on the structural, optical, and electronic properties of these materials and on the structure-property relationships. The samples were prepared using SnCl_4_ as tin source and ammonia as precipitating agent and N source. The Sn/Ti molar content was varied in the 0–20% range and different post-treatments were used to promote sample crystallinity: either a calcination in oxidizing environment or a prolonged, low temperature hydrothermal treatment. In this respect, literature studies report shorter hydro/solvothermal treatments (24–72 h) for TiO_2_ growth, but, in much harsher conditions [29,30,31,32], while flash treatments (few hours) are only reported for the preparation of TiO_2_ crystal seeds [33,34]. In the present case, a prolonged hydrothermal treatment was adopted for promoting crystal growth in less demanding conditions, with notable advantages in terms of safety concerns and required instrumentation. It was observed that very different structural and optical properties can be obtained, depending on the post-treatment and on the quantity of Sn.

## 2. Materials and Methods

During syntheses, doubly-distilled water passed through a Milli-Q apparatus and analytical grade reagents and solvents (Sigma–Aldrich, Milan, Italy) were used. Sn-promoted TiO_2_ samples were prepared according to a previously reported sol-gel procedure [20,28]. Titanium(IV) isopropoxide (TTIP, 37.6 mmol), along with an appropriate amount of SnCl_4_∙5H_2_O, was mixed with 2-propanol. Subsequently, a NH_4_OH aqueous solution was slowly dripped into the reaction flask while stirring at 300 rpm. The adopted molar ratios were 1:20:0.5:100 for TTIP:2-propanol:NH_4_OH:H_2_O, and the Ti:Sn molar ratio was varied in the 0–20% range. The reaction mixture was then stirred at 60 °C for 90 min., and then dried overnight at 80 °C and ambient pressure. The resulting powder was washed several times by centrifugation-resuspension cycles and then dried in oven. For the sake of comparison, the TiO_2_ reference samples were also prepared with the same method without the addition of Sn species.

Two different post-synthetic treatments were compared in order to promote the sample crystallinity: a hydrothermal treatment in mild conditions and a conventional calcination procedure. For the hydrothermal treatment, 1.5 g of xerogel was suspended in water (50 mL) and then transferred into a 100 mL Teflon-lined stainless-steel autoclave. The reaction mixture was heated at 100 °C for 170 h. The calcined samples were instead treated at 400 °C under O_2_ flux (9 NL·h^−1^) for 6 h.

The samples were labeled as TiSnx_y, where x is the Sn/Ti molar ratio, while y is a letter indicating the type of post-synthetic treatment (H for hydrothermal and C for calcination). Similarly, reference TiO_2_ samples with no Sn content were named Ti_H and Ti_C. Commercial pure TiO_2_ polymorphs (Sigma–Aldrich) were also used as benchmarks. The SnO_2_ samples were also prepared as reference: both commercial (Sigma–Aldrich) and synthesized oxides were adopted for this purpose. The latter was prepared by a previously reported procedure [35]; in brief, it was synthesized by precipitation starting from a 0.1 M SnCl_4_∙5H_2_O aqueous solution and using urea as precipitating agent (Sn:urea molar ratio 1:7) at 90 °C for 8 h; the precipitate was washed then treated in a Teflon-lined stainless-steel autoclave at 130 °C (SnO_2__H); another sample was subjected to a final calcination at 400 °C in O_2_ flux (SnO_2__C).

The optical properties of the samples were determined by diffuse reflectance spectroscopy (DRS). The spectra were recorded in the 250–800 nm range on a UV-vis spectrophotometer (Shimadzu UV-2600, Shimadzu Italia S.r.l., Milan, Italy) that was equipped with an integrating sphere. Reflectance curves were converted to absorptivity by applying the Kubelka–Munk function, F(R):

F(*R*) = (1 − *R*)^2^/2*R* = *K*/*S*

where *R* is the reflectance from a thick layer of powder, *K* is the absorptivity, and *S* is the scattering factor.

High resolution transmission electron microscopy (HRTEM) images were collected using a JEOL 3010–UHR microscope (JEOL (ITALIA) S.p.A., Milan, Italy), working at a 300 kV acceleration potential and equipped with a LaB_6_ filament. Micrographs were acquired using an Ultrascan 1000 camera and image processing was carried out using the Gatan Digital Micrograph 3.11.1 software. Before analysis, the sample powders were dry dispersed onto copper grids coated with “lacey” carbon film.

Fourier transform infrared (FT-IR) spectra were acquired on a PerkinElmer Spectrum 100 FT-IR spectrometer (PerkinElmer Italia S.p.A., Milan, Italy) working in attenuated total reflectance.

XAS spectra were measured at the XAFS beamline [36] operating at the Elettra synchrotron radiation facility in Trieste, Italy. The spectra were acquired at room temperature at the Ti K-edge and at the Sn L_2,3_-edges in transmission mode. The ring current and energy were 200 mA and 2.4 GeV, respectively. A Si(111) double crystal monochromator was used, ensuring high-order harmonic rejection by the de-tuning of the second crystal and a water cooled Pt-coated silicon mirror was used to obtain vertical collimation of the beam. For the measurements, a proper amount of sample (as to give a unit jump into the absorption coefficient) was mixed with boron nitride and then pressed into a pellet. The signal extraction was performed with the Athena code, belonging to the set of interactive programs IFEFFIT [37,38]. The pre-edge background was fitted by means of a straight line, while the post-edge by a cubic spline. All of the spectra were then normalized at unit absorption well above the edge, where the EXAFS oscillations were no more visible.

## 3. Results and Discussion

### 3.1. Structural Properties of the TiO_2_ Matrix

**Figure 1:**
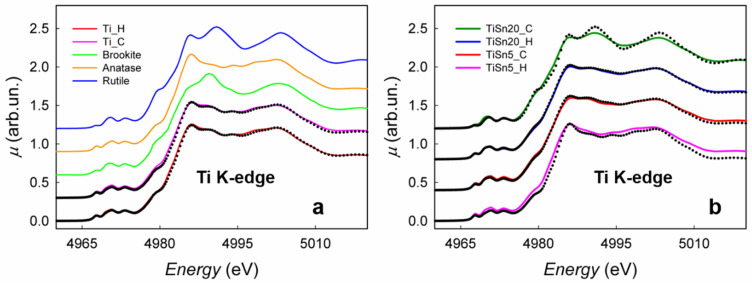
(**a**) X-ray absorption (XAS) spectra at the Ti K-edge of rutile, anatase, brookite, Ti_C, and Ti_H. The linear combination fits for Ti_C and Ti_H are shown by the correspondent black dotted lines. (**b**) XAS spectra at the Ti K-edge of TiSn20_C, TiSn20_H, TiSn5_C, and TiSn5_H. The black dotted lines in correspondence with TiSn20_C and TiSn5_H are the reference spectra of rutile and anatase, shown for the sake of comparison, while the black dotted lines in correspondence with TiSn5_C and TiSn20_H are the result of the linear combination fit, as listed in Table 1.

X-ray absorption spectra were acquired both at the Ti K-edge and at the Sn L_2,3_-edges in order to obtain a complete picture of the electronic and structural properties of the Sn-copromoted TiO_2_ nanomaterials. Here, we warn the reader that structural probes, such as XAS and TEM, are only indirectly sensitive to the presence of N in the structure, which, on the contrary, may affect in a relevant way the optical properties. Ti K-edge spectra were first measured on the reference materials, as shown in Figure 1a. TiO_2_ can exist in three possible structural modifications, namely anatase, rutile, and brookite, which mainly differ in the connectivity of the TiO_6_ octahedral units. While, for bulk samples, rutile is the most stable phase at ambient pressure and room temperature, the synthesis of nanometric TiO_2_ can also lead to the stabilization of brookite and anatase. Ti K-edge spectra result from the transition from filled 1*s* orbitals to empty 4p Ti orbitals; the different connection of octahedra leads to different binding energies and density of empty states, with the result that the three Ti K-edge spectra of rutile, anatase, and brookite are different and easy to discriminate. The spectra of the Ti_H and Ti_C, corresponding to TiO_2_ with no Sn content and synthesized through hydrothermal synthesis (H) or calcination (C), closely resemble each other, and they clearly show the presence of different TiO_2_ phases; for a quantification, a linear combination fit was performed, starting from the rutile, anatase, and brookite spectra. For both samples, the best fit showed the presence of 76(2)% anatase and 24(1)% brookite. However, it should be noted that, in the case of nanocrystalline particles of small dimensions (as in the present case, see the HRTEM results below), a notable broadening of the main XAS peaks is expected, and this can lead to a certain degree of error in the quantification of phases.

Figure 1b shows the XAS spectra of TiSn20_C, TiSn5_C, TiSn20_H, and TiSn5_H at the Ti K-edge.

The derivative spectra of all the samples were also calculated and compared to the derivates of the standard spectra, in order to search for possible shifts of the edge energy (see Figure S1a); the precise correspondence of all the features in the derivatives allows for excluding the presence of Ti in oxidations states different from Ti (IV), at least within the sensitivity limits of the technique (±5%). The N,Sn-copromoted TiO_2_ nanoparticles described here have a radius of ca. 5 nm, meaning that ca. 10% of Ti atoms are at the surface. This figure is twice the sensitivity of ca. 5% of XAS, which is not an error in the data analysis, but rather an intrinsic sensitivity limit of the technique. This means that the contribution of the Ti atoms at the surface is rather relevant for what concerns the XANES.

The spectrum of TiSn20_C presents all of the structures corresponding to rutile, shown for the sake of comparison as a black dotted line. As expected, and as pointed out before, while the peaks lie in the same position of the reference spectrum, they show a notable broadening; however, it can be safely stated that TiSn20_C has a pure rutile structure, notwithstanding the low calcination temperature here adopted (400 °C). Doping with tin seems to favor the formation and, therefore, the stabilization of rutile, which is isostructural with SnO_2_ in its most stable modification, cassiterite. In this respect, Okajima et al. reported DFT calculations showing that Sn(IV) substitutional doping in the TiO_2_ structure reduces the formation energy of rutile with respect to anatase, hence favoring the formation of the rutile [22]. Therefore, in the present case, the formation of pure rutile in the TiSn20_C sample can be related to its high Sn content coupled to the calcination procedure. When the Sn/Ti ratio is lower, i.e., 5%, and for the same synthetic method (TiSn5_C sample), the best fit shows a mix of anatase, brookite, and rutile. In this case, the quantity of tin seems to be too low to induce the neat formation of rutile TiO_2_. The same happens when the samples are synthesized via the milder hydrothermal procedure, even when the Sn/Ti ratio is 20%. In the case of TiSn5_H, no trace of rutile is found, and all of the peaks clearly coincide with the anatase reference spectrum (shown for comparison in Figure 1b as the black dotted line). A reliable phase quantification for TiSn5_C and TiSn20_H was possible via a linear combination fit starting from TiSn20_C and TiSn5_H, which account for Sn-copromoted titania with pure rutile structure and with pure anatase structure, respectively. As an example, the linear combination fit for TiSn20_H is better shown in Figure S1c, together with the corresponding residual; all of the residuals (experimental data minus fit) are shown for all the samples in Figure S1d, while panel b shows the difference between the spectra of TiSn20_C and TiSn5_H and the standard spectra of rutile and anatase, respectively. In this last case, it can be noted that all of the differences are due to the nanometric size of the samples with respect to the standards, as pointed out before. Concerning the experimental fits, it can be noted that in all cases the residual is low and negligible, thus confirming the good quality of the fitting procedure.

TiO_2_ in TiSn20_H is a mix of anatase and rutile, despite the relatively high quantity of tin, as shown in Table 1. This indicates that probably, in this Sn/Ti ratio, the hydrothermal synthesis prevents most of tin atoms to penetrate in the lattice, leaving SnO_2_ confined at the surface of the nanoparticles. This hypothesis is supported by previous XPS/EDX studies [28], showing substantial surface segregation of tin species for hydrothermal samples, in agreement with a much lower bulk penetration of the heteroatoms during the low temperature post-treatment with respect to calcined samples. In Table 1, the phase quantification that is obtained by XAS is compared to previous results obtained from XRD measurements (Table 1, column 5) [28]. When considering the different sensitivity of the two techniques to local and long-range order, respectively, the agreement is rather satisfactory.

**Figure 2:**
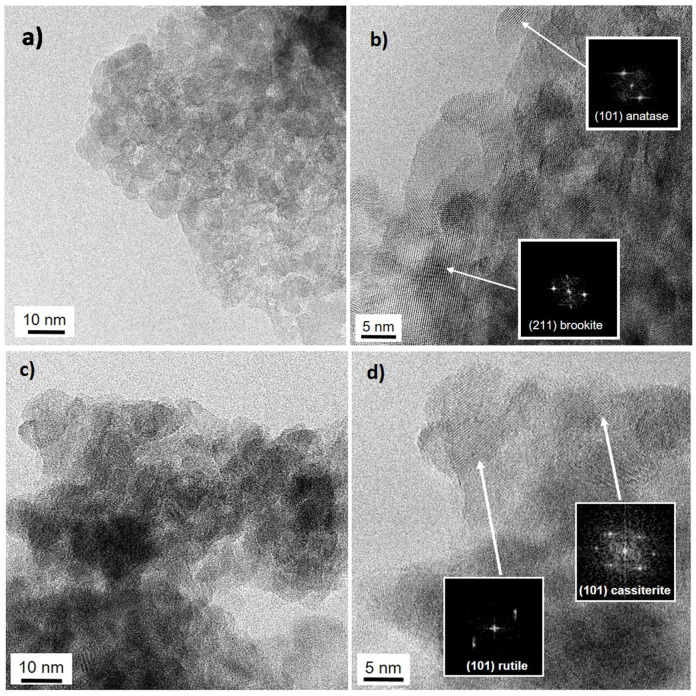
TEM images of (**a**,**b**) TiSn5_C and (**c**,**d**) TiSn20_C. Scale bars are 10 nm (**a**,**c**) and 5 nm (**b**,**d**). Insets: electron diffraction patterns of the detected polymorphs.

TEM images support the phase composition that has been determined by XAS analyses. Figure 2 reports representative (HR)TEM images of TiSn5_C and TiSn20_C. The samples are characterized by a good degree of crystallinity and exhibit pseudo-spherical crystallites, generally smaller than 10 nm in size, which is in good agreement with the values determined by Scherrer analysis of diffractograms [20,28]. Figure 2a,b confirm that, when a lower tin content is introduced, TiO_2_ is mostly in the anatase form (d_101_ = 0.339–0.342 nm), with some evidence of the brookite polymorph (d_211_ = 0.291–0.294 nm). Moreover, at this low Sn content, segregated cassiterite SnO_2_ is only seldom recognizable, as it will also be discussed on the grounds of Sn L_2,3_-edges XAS spectra. In the case of TiSn20_C (Figure 2c,d), TiO_2_ is mostly present as the rutile polymorph, in close agreement with the Ti K-edge XAS findings. Along with rutile TiO_2_ (d_101_ = 0.246–0.249 nm), highly dispersed cassiterite SnO_2_ is also appreciable at this high Sn content, both on the basis of interplanar distances (d_101_ = 0.260–0.263 nm) and of the electron diffraction patterns (Figure 2d). In this respect, it should be noted that previous XRD investigations [20,28] showed no well-defined peaks that were related to SnO_2_ phases, possibly due to the highly dispersed nature and low content of segregated cassiterite Moreover, because cassiterite and rutile are isostructural, their reflections are substantially overlapped in the XRD pattern, thus preventing easy distinction between the phases. The localization of Sn species in the samples will be further discussed on the grounds of XAS spectra at the Sn L_2,3_-edges.

### 3.2. Tin Location in the Oxide

**Figure 3:**
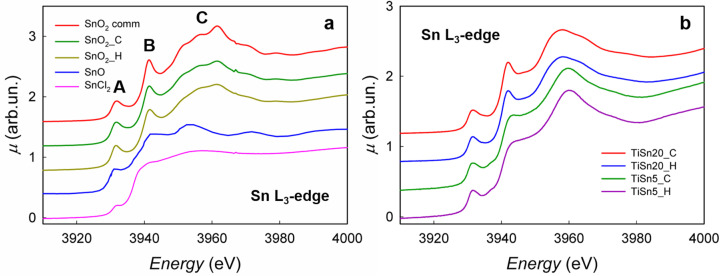
(**a**) XAS spectra at the Sn L_3_-edge of commercial SnO_2_, SnO, and SnCl_2_, and of SnO_2__C and SnO_2__H samples. (**b**) XAS spectra at the Sn L_3_-edge of TiSn20_C, TiSn20_H, TiSn5_C, and TiSn5_H.

XAS spectra were acquired at the Sn L_2,3_-edges for a better understanding of the role of Sn on the structural and electronic properties of our samples. The Sn L_3_-edge spectra of some reference compounds were first acquired and they are shown in Figure 3a.

The spectra of SnO_2_ synthesized via calcination (SnO_2__C) and hydrothermal procedure (SnO_2__H) show a close resemblance to commercial SnO_2_, despite some broadening of the main peak (White Line, WL). In XAS spectra, peaks in the close proximity of the edge can be ascribed to a specific electronic transition to empty states that are projected on the photoabsorber. The L_3_-edge is due to the transition from filled 2p_3/2_ Sn states to empty Sn orbitals of either 5s or 5d nature. In particular, the peaks that are indicated as A and B in Figure 3 are due to the transition 2p_3/2_ → 5s_1/2_, while the whole region that is indicated as C is due to transition to empty 5d_5/2_ orbitals [26]. In SnO_2_, tin has electronic configuration [Kr]4d^10^5s^0^5p^0^: the 5s orbitals, being empty, are accessible to the photoelectron, thus resulting in two well visible peaks, A and B. The exact origin of these two peaks has yet to be clarified as, in principle, the 2p_3/2_ → 5s_1/2_ transition should give rise to just one peak. We here offer a tentative attribution by considering the similar situation of the Ce L_3_-edge in CeO_2_ [39,40], although a precise interpretation has to be supported by full calculations that are outside the aims of the present paper. In CeO_2_, the L_3_-edge is characterized by a two peak structure, while the 5d^0^ electronic configuration of Ce(IV) would suggest the presence of just one peak. Final state effects, and the covalence of the Ce-O bond in CeO_2_, allow for transitions to the empty 5d states of the 4f^0^ and of the 4f^1^L electronic configurations, where L represents a hole in the ligand states [31], thus giving rise to the two peak structure. Here, we can also note that the Sn-O bond in SnO_2_ has a large covalent character. Thus, the Sn 5s and 5p states are expected to show a high degree of hybridization with the O 2*p* states. Correspondingly, ligand electrons are transferred in the 5s states, giving rise to the additional electronic configuration 5s^1^L, where L represents a hole in the ligand states. In analogy with CeO_2_, we can then attribute peak A to the 2p_3/2_ → 5s_1/2_ transition of the s^0^ electronic configuration, and peak B to the 2p_3/2_ → 5s_1/2_ of the 5s^1^L electronic configuration. In SnO, the electronic configuration of tin is [Kr]4d^10^5s^2^5p^0^, so it would be expected to have a negligible intensity of peaks A and B; however, probably due to partial hybridization between s and p orbitals, these peaks show a lower intensity, but they are still visible in the spectrum (blue line in Figure 3).

**Figure 4:**
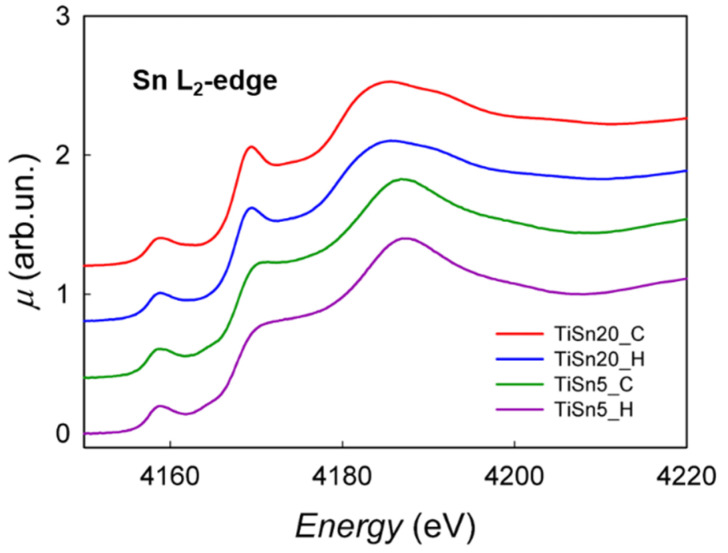
XAS spectra at the Sn L_2_-edge of TiSn20_C, TiSn20_H, TiSn5_C, and TiSn5_H.

Figure 3b shows the spectra of TiSn20_C, TiSn20_H, TiSn5_C, and TiSn5_H at the Sn L_3_-edge, while the spectra at the L_2_ edge are reported in Figure 4. The spectra of TiSn20_C and TiSn20_H show a close resemblance to each other and are similar to the spectra of SnO_2__C and SnO_2__H, despite minor differences in the white line. This can be rationalized when considering that TiSn20_C presents a sizable rutile content, which provides a neighboring environment that is similar to cassiterite; in addition, HRTEM micrographs showed that part of SnO_2_ remains segregated as cassiterite. Unfortunately, it is impossible to discern from XAS the portion of segregated SnO_2_ due to the similar neighboring environment or rutile and cassiterite, thus preventing any precise quantification. Concerning TiSn20_H, the quantity of rutile is lower (28%), thus indicating that most of SnO_2_ does not enter the structure. This is in agreement with our observation at the Sn L_3_-edge.

The XAS spectra of TiSn5_C and TiSn5_H have a profile that is completely different from samples with 20% Sn/Ti ratio, clearly showing that, in this case, the chemical surroundings of Sn are not those that are typical of cassiterite. When considering that, as demonstrated by the Ti K-edge analysis, Ti is mostly present as anatase TiO_2_, it is reasonable to suppose that in this case Sn is not segregated (as also observed by TEM analysis), but it is a substitutional dopant in the TiO_2_ structure, with the chemical surrounding typical of anatase/brookite, leading to the characteristic spectrum profile that is shown in Figure 3b. When considering a complete insertion of Sn in the TiO_2_ structure, we could, in principle, say that 5% (as molar ratio) of Ti^4+^ is affected by disorder due to the substitution with Sn^4+^. Moreover, the most striking feature of the two spectra of TiSn5_C and TiSn5_H is the notable reduced intensity of the peak indicated as B in Figure 3a. The lower intensity of this peak indicates that the 5s orbitals are partially filled, as mentioned above: this points towards the fact that Sn might be partially present in a reduced Sn^2+^ state. Moreover, in agreement with the previous attribution, peak B is strongly depressed by the addition of extra electrons in the 5s^1^L electronic configuration, while peak A, due to the 5s^0^ electronic configuration, still retains a considerable intensity. A subtraction procedure was applied to isolate peak B in order to quantify the amount of reduced tin, as it is shown in the Supplementary Materials (Figure S2). The areas of peak B for the different samples was then obtained (see Supplementary Materials, Table S1), and the fraction of reduced tin was obtained by $\;1-\left(A_{S}-A_{\mathrm{SnO}}\right)/\left(A_{{\mathrm{SnO}}_{2}}-A_{\mathrm{SnO}}\right)$, where $A_{S}$, $A_{\mathrm{SnO}}\;$ and $A_{{\mathrm{SnO}}_{2}}$ are the areas of peak B in each sample, SnO and SnO_2_, respectively. Table S1 shows the results. The reader is warned that, mainly due to the arbitrariness of the background subtraction procedure, the values that are shown in Table S1 are to be considered as semi-quantitative. It is reasonable to suppose that Sn^2+^ is still a substitutional dopant in the TiO_2_ structure; in fact, intrinsic defects in SnO_2_ give rise to oxygen vacancies that are compensated by substitutional Sn(II) on the Sn(IV) sites [41], while aliovalent self-doping of SnO_2_ with Sn(II) is a commonly used tool for improving the electrical properties, which are relevant for the applications of SnO_2_ in gas sensing [42]. This means that Sn(II) can easily replace Sn(IV) in the SnO_2_ structure. Thus, substitutional doping of Sn(II) in the TiO_2_ structure can be deemed as possible, given the close similarity in ionic radii of Ti(IV) and Sn(IV) and the fact that SnO_2_ has the rutile structure.

Figure 4 shows the XAS spectra at the Sn L_2_-edge; in this case, the transition is from filled 2p_1/2_ orbitals to empty s or d orbitals above the Fermi level. It can be seen that the profile is analogous to that found for the L_3_-edge, which confirms the presence of tin in a partial reduced state for the TiSn5_C and TiSn5_H samples.

It is worth noting that evidence of tin in a partially reduced state is found not only for TiSn5_H, which underwent a mild hydrothermal treatment, but also for TiSn5_C, which was, in turn, calcined in O_2_ at 400 °C. This could be explained by a combined effect of ammonia in the synthetic procedure, which creates a reducing environment, and of the N-doping, which could concur to stabilize the formation of Sn(II) (*vide infra*).

### 3.3. Optical Properties

**Figure 5:**
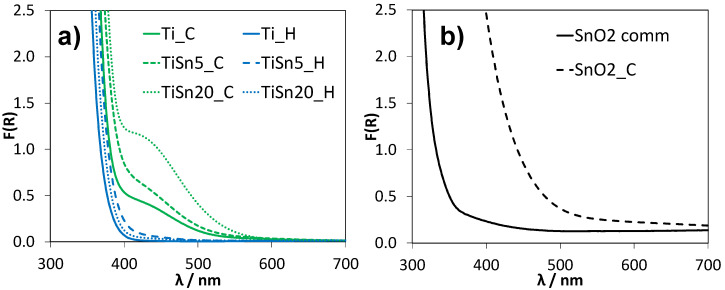
Normalized diffuse reflectance spectra (Kubelka–Munk function vs. wavelength) of pristine and Sn-promoted TiO_2_ samples (**a**) and SnO_2_ samples (**b**).

The role of N doping in the samples, as mentioned above, can be better evidenced by the study of the optical properties. The optical properties of Sn-promoted TiO_2_ samples present notable differences, depending on the Sn/Ti molar content and on the post-treatment. The normalized diffuse reflectance spectra (Kubelka–Munk function vs. wavelength) are shown in Figure 5a. The spectra of Ti_H and Ti_C are also reported for the sake of comparison.

Figure 5a shows a marked difference in terms of visible light absorption between hydrothermal and calcined TiO_2_ samples, irrespective of the amount of Sn introduced. While samples from hydrothermal treatment present the classical light absorption of TiO_2_ semiconductors, with an absorption edge at ca. 390 nm, the calcined samples present a marked absorption in the visible region. In particular, both undoped and Sn-promoted TiO_2_ samples upon calcination show, beside the light absorption in the UV region characteristic of TiO_2_, a light absorption centered at about 450 nm that imparts a yellowish color to the powder. This optical feature can be attributed to the occurrence of defects giving rise to intragap electronic states. Similar optical properties have been reported in the case of N-doped TiO_2_ [43] and attributed to intragap states due to N interstitial doping within the TiO_2_ lattice [44]. It should be noted that N species (ammonia) was added during the synthesis of all TiO_2_ samples. Thus, it can be assumed that the calcination step promotes the diffusion of N species in the TiO_2_ structure, giving rise to N-doping and, consequently, to visible light absorption. This attribution is confirmed by DR spectra of Sn-doped TiO_2_ samples that were prepared using the same synthetic procedure, followed by calcination, but without the addition of nitrogen containing species (Figure S3): the absence of the absorption component in the visible region for the Sn-doped TiO_2_ sample supports the attribution of this feature to N-doping. On the contrary, the hydrothermal treatment appears to be unsuitable for introducing the N species within TiO_2_, leading to optical properties mirroring undoped anatase-brookite TiO_2_ [45], and supported by the comparison with Figure S3. A lower diffusion of N species in the case of hydrothermal samples is also supported by FTIR evidence (Figure S4), showing, for these samples, irrespective of the presence of Sn, the occurrence of surface N species: the peak at ca. 1430 cm^−1^, not appreciable for calcined samples, can be ascribed to surface NH^4+^ species [46,47,48].

The optical properties of SnO_2_ samples were also considered to better understand the effect of tin doping in the TiO_2_ samples. Figure 5b reports the DR spectra of SnO_2__C with respect to a commercial SnO_2_ powder: both of the samples exhibit absorption curves that are characteristic of semiconductor materials; however, the position of the absorption edge varies greatly between the two samples. While the commercial oxide shows a light absorption only in the UV region, in good agreement with literature band gap values for SnO_2_ [18,19], the SnO_2__C sample presents a red-shifted absorption edge that leads to a marked visible light absorption up to ca. 500 nm. It is noteworthy that N species were added also during SnO_2__C synthesis. In this respect, the occurrence of N-doping in pure SnO_2_ has been investigated both theoretically [47,49,50] and experimentally [51,52,53]. Theoretical calculations and experimental evidence support a preferential occurrence of substitutional doping of nitrogen atoms on O sites in SnO_2_, leading to intragap acceptor levels due to N species [47,49,54]. These intragap states can red-shift the absorption edge of SnO_2_ [50], as observed in the present sample. Moreover, experimental investigations have shown evidence of oxygen vacancies or reduced SnO_2_ occurring along N-doping [51,52]. Interestingly, previous studies have shown that the incorporation of N atoms on the oxygen sites of SnO_2_ is significantly enhanced by the high temperature oxidation and the optimal temperature is about 400 °C [53]. A contribution of N-doped SnO_2_ to the light absorption of N,Sn-copromoted TiO_2_ samples showing segregated cassiterite could occur, especially for calcined samples (*vide infra*).

**Figure 6:**
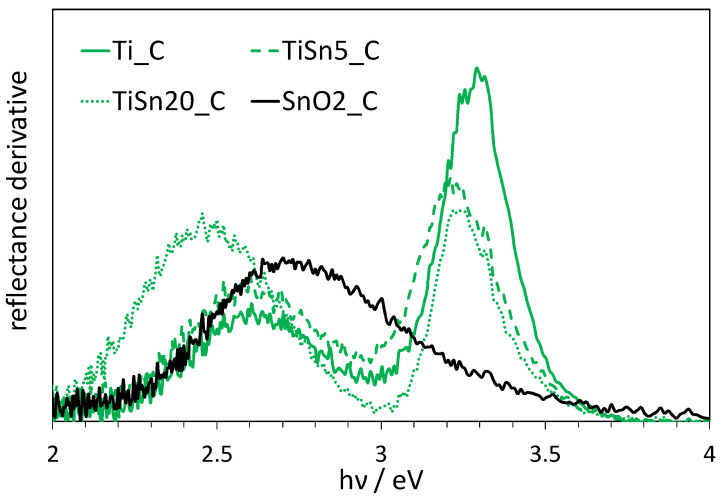
First derivative of reflectance spectra of calcined samples as a function of radiation energy (in eV).

To better clarify this aspect, the light absorption features of calcined samples were further compared by means of first derivative reflectance spectra (Figure 6).

TiSnx_C samples show a red shift of ca. 0.1 eV in the main absorption edge, related to the main semiconductor band gap, with respect to the reference Ti_C, as clearly appreciable in Figure 6. In this respect, it should be noted that TiSnx_C samples exhibit increasing amounts of rutile: the lower band gap of this polymorph (3.0 eV) could partially explain the observed red shift. However, a larger shift is observed for the TiSn5_C sample, which has a lower rutile content than TiSn20_C. Moreover, a similar red shift is also observed in the case of the TiSn5_H sample (Figure S5), which contains no rutile. While rutile content can surely cause a red shift of the absorption edge, this cannot be the sole reason behind the observed shift for TiSn5_x samples, because of the opposite trends in rutile content and observed red shift as a function of Sn-content. Thus, on the grounds of Sn L_2,3_-edge findings, it can be proposed that the observed shift in the absorption edge of TiSn5_C and TiSn5_H can be related to Sn(II) states, also in agreement with previous reports [3] and as supported by DFT calculations of electronic structure [3].

The second component centered at ca. 2.60 eV, observed in both TiSn5_C and Ti_C samples, can be attributed to intragap states that are related to N-doping, on the grounds of previous characterization of Ti_C sample [43,44,45,55] and as supported by the comparison with Sn-doped sample without N species (Figure S5). This component appears to be slightly enhanced in the N,Sn-copromoted sample, which suggests a synergistic effect of Sn(II) species with respect to N-doping. In this respect, it has been previously reported that nitrogen incorporation is favored by the presence of TiO_2_ lattice defects, such as oxygen vacancies [56,57]. *Vice versa*, N-doping has also been reported to favor the occurrence of oxygen vacancies [58], which might, in turn, favor the formation of Sn(II) species.

It should be noted that a quantification of bulk nitrogen species is a difficult task, since the N content is at least one order of magnitude lower than the nominal content because of a loss of N species during calcination [43,44,45]. As a result, the quantification of the actual nitrogen bulk content is quite challenging, since techniques, such as XPS, provide a surface sensitive information, which is thus greatly affected by N-containing atmospheric contaminants [59].

In the case of the TiSn20_C sample, the absorption in the visible region is further promoted, but it is red shifted with respect to Ti_C and TiSn5_C. As this sample is composed of rutile TiO_2_ and cassiterite SnO_2_ (as supported by XAS and HRTEM findings), on the grounds of the SnO_2__C optical properties and of literature studies of N-doped rutile [60,61], the observed light absorption of the TiSn20_C samples seems to be mainly compatible with nitrogen interstitial doping in the TiO_2_ structure. No evidence of light absorption by N-doped SnO_2_ is appreciable, since the visible light component of the TiSn20_C sample is shifted of ca. 0.3 eV with respect to the SnO_2__C light absorption. The occurrence of segregated cassiterite seems to contribute to light absorption mainly in an indirect way. The markedly enhanced visible light absorption could be related to the higher defectivity that is associated with the highly dispersed SnO_2_ phase in the sample. Although no Sn(II) could be detected in the samples with 20% Sn/Ti ratio, a consideration should be made: XAS provides an averaged information on all the Sn atoms in the samples. The signal is not expected to drastically change if only few centers exhibit a different environment. As in TiSn20_x samples, most of tin is segregated as cassiterite; this component is mostly responsible for the observed signal at the expense of any dopant atoms within the TiO_2_ structure.

## 4. Conclusions

In this work, a combined X-ray absorption spectroscopy investigation was carried out on N,Sn-copromoted TiO_2_ nanoparticles at both the Ti K-edge and Sn L_2,3-_edges, with the aim of correlating the material structural and the optical properties. In the samples, the Sn/Ti molar content was varied in the 0–20% range and different post-treatments (calcination and low temperature hydrothermal treatment) were considered.

Irrespective of the type of post-treatment adopted, when the Sn/Ti ratio is high (20%), part of tin remains segregated as SnO_2_ at the surface of the nanoparticles, as shown by HRTEM and Sn L_2,3_-edge findings. However, part of tin enters the TiO_2_ structure, more so in calcined samples, inducing the formation of TiO_2_ in the rutile phase, as supported by HRTEM and Ti K-edge XAS evidence. Hydrothermally treated samples seem to show a lower diffusion of heteroatoms (Sn, N) within TiO_2_, which could be inferred by the higher anatase content, surface N species, and limited visible light absorption.

When the Sn/Ti ratio is lower (5%), the quantity of tin is not enough to induce the formation of pure rutile, also in the case of calcined samples, so TiO_2_ remains mainly an anatase/brookite mixture. At this lower Sn content, tin is not segregated as SnO_2_, but it is an effective dopant in the TiO_2_ structure, as shown by Sn L_2,3_-edge XAS; moreover, there is evidence of the formation of Sn in a partially reduced state. This is possibly related to ammonia used in synthesis and the N-doping, which might stabilize the formation of Sn(II). It is worth noting that, to the best of our knowledge, this is the first experimental observation of reduced tin states in TiO_2_ samples that were treated in highly oxidizing conditions and at high temperature. The reduced Sn species are responsible for a red shift of the absorption edge of samples with lower Sn content.

On the other hand, the best optical properties were found for the calcined sample with Sn/Ti ratio that was equal to 20%. It should be noted that TiO_2_ promoted with only Sn species has no enhanced light absorption in the visible, whereas N,Sn-copromoted, calcined TiO_2_ samples present an increased visible absorption with respect to N-doped TiO_2_, pointing towards synergistic effects between N and Sn on the optical properties of TiO_2_ photocatalysts. This phenomenon can be the result of the combined effect of the calcination process, which enhances the diffusion of N species within the lattice, and of the defectivity induced by tin species, including highly dispersed SnO_2_, which promotes the incorporation of N in the TiO_2_ structure.

## Figures and Tables

**Table 1:** Anatase, brookite, and rutile content (expressed as percentage) and associated error (in brackets) in the Sn-promoted TiO_2_ samples derived from the linear combination fit of Ti K-edge XAS spectra. Phase composition (A: anatase, B: brookite, R: rutile) determined by X-ray diffraction (XRD) is also reported for the sake of comparison from Ref. [28].

Sample	Phase Content From XAS			Phase Composition From XRD
	Anatase (%)	Brookite (%)	Rutile (%)	
Ti_C	76(2)	24(1)	/	66A–34B
Ti_H	76(2)	24(1)	/	80A–20B
TiSn20_C	/	/	100	100 R
TiSn20_H	55(2)	/	45(2)	30A–70R
TiSn5_C	58(3)	14(7)	28(6)	78A–22B
TiSn5_H	100	/	/	96A–4B

## Supplementary Materials

The following are available online at https://www.mdpi.com/2079-4991/10/6/1224/s1, Figure S1: Derivative spectra of all the samples and standards at the Ti K-edge, residuals (experimental data minus fit lines) for all the samples, Figure S2: Isolation procedure of peak B in the case of TiSn5_C sample, Figure S3: DR spectrum of a calcined Sn-doped TiO_2_ sample without N-doping, Figure S4: FTIR spectra of calcined and hydrothermal samples, Figure S5: First derivative of reflectance spectra of hydrothermal samples as a function of radiation energy (in eV), Table S1: Quantification of Sn(II) in TiSn5_C and TiSn5_H.
