# Supplementary material for: Role of Synthetic Parameters on the Structural and Optical Properties of N,Sn-Copromoted Nanostructured TiO2: A Combined Ti K-Edge and Sn L2,3-Edges X-ray Absorption Investigation
Source: Nanomaterials (Basel). 2020 Jun 23;10(6):1224. doi: 10.3390/nano10061224 (PMC7353116; doi:10.3390/nano10061224)
Supplement: Supplementary file 1 [file nanomaterials-10-01224-s001.pdf]

Role of synthetic parameters of the structural and optical properties on N,Sn-copromoted nanostructured TiO_2: a combined Ti K-edge and Sn $L_{2,3}$-edges X-ray absorption investigation

Martina Fracchia [1] *, Paolo Ghigna [1,2], Alessandro Minguzzi [2,3], Alberto Vertova [2,3], Francesca Turco [4], Giuseppina Cerrato [4] and Daniela Meroni [2,3] *

[1] Department of Chemistry, Università degli Studi di Pavia; martina.fracchia02@universitadipavia.it, paolo.ghigna@unipv.it
[2] Consorzio Interuniversitario per la Scienza e Tecnologia dei Materiali (INSTM), via Giusti 9, 50121 Florence, Italy
[3] Department of Chemistry, Università degli Studi di Milano, via Golgi 19, 20133 Milan, Italy; daniela.meroni@unimi.it; alessandro.minguzzi@unimi.it; alberto.vertova@unimi.it
[4] Department of Chemistry and NIS, Inter-departmental Center, Università di Torino, Via Giuria 7, 10125 Torino, Italy; giuseppina.cerrato@unito.it; francesca.turco@unito.it
* Correspondence: martina.fracchia02@universitadipavia.it, daniela.meroni@unimi.it

SUPPLEMENTARY MATERIALS

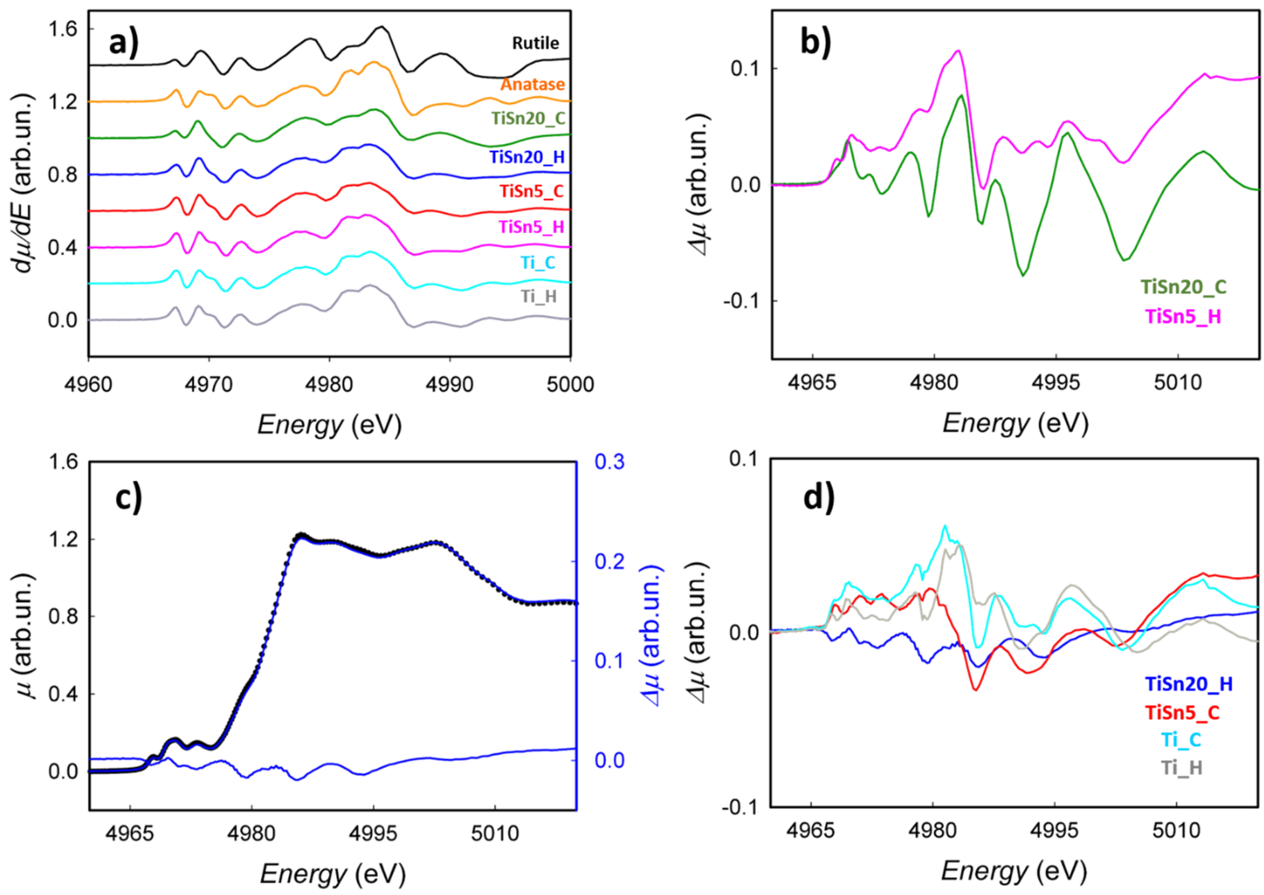

Figure S1. (a) Derivative spectra of all the samples and standards at the Ti K-edge. (b) Difference spectra obtained between the spectrum of TiSn20_C and the spectrum of rutile (green line) and between the spectrum of TiSn5_H and the spectrum of anatase (magenta line). (c) XANES spectrum of TiSn20_H at the Ti K-edge (solid blue line) and linear combination fitting (black dotted lines). The residual (experimental data minus fitting) is shown by the blue line. (d) Residuals (experimental data minus fitting) calculated for TiSn20_H, TiSn5_C, Ti_C and Ti_H.

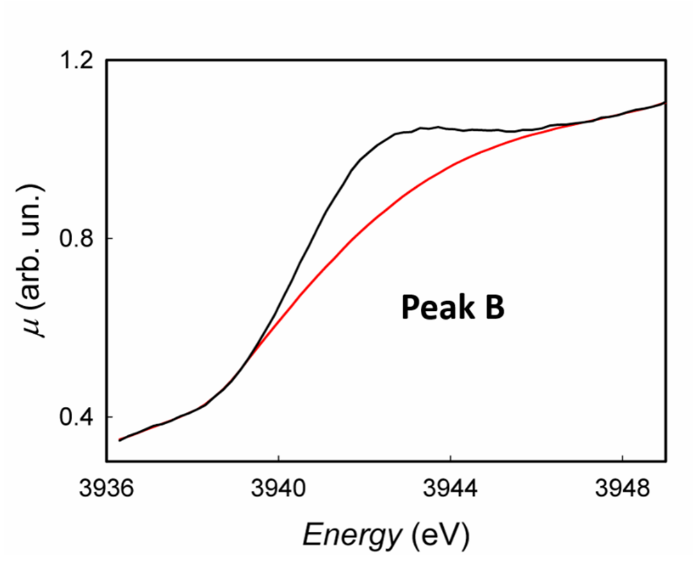

Figure S2. Isolation procedure of peak B in the case of TiSn5_C sample. The red line is the background function, obtained through the Statistics-sensitive Non-linear Iterative Peak-clipping (SNIP) algorithm, a background approximation that provides reliable treatment of fluctuations[1].

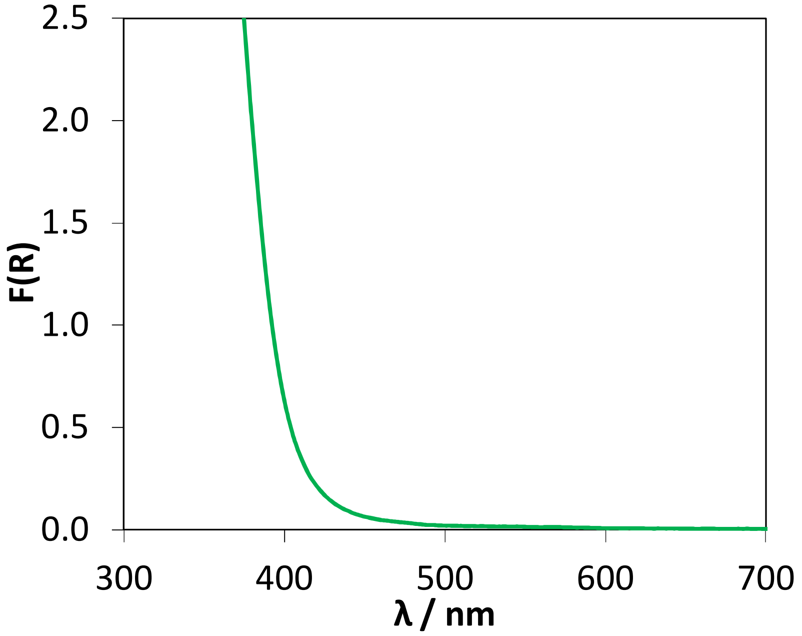

Figure S3. DR spectrum of a calcined Sn-doped TiO_2 sample without N-doping.

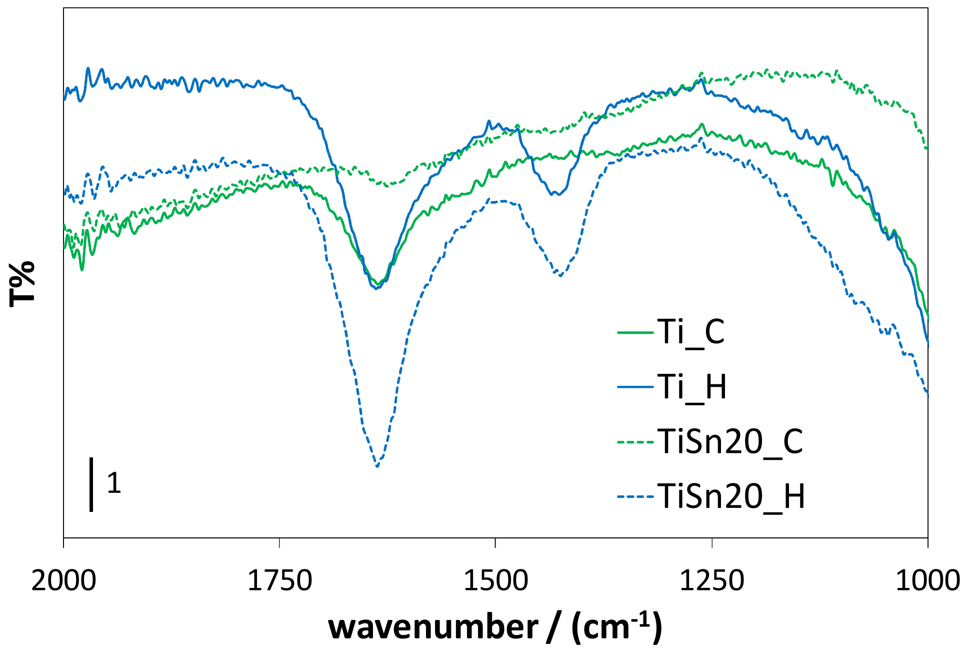

Figure S4. FTIR spectra of calcined and hydrothermal samples.

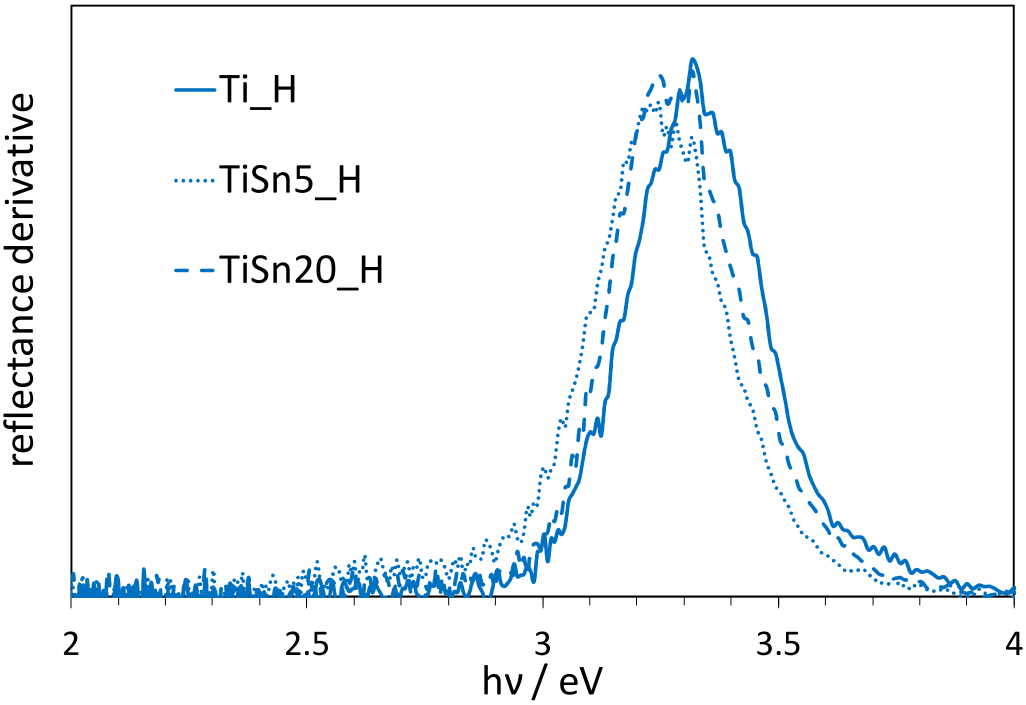

Figure S5. First derivative of reflectance spectra of hydrothermal samples as a function of radiation energy (in eV).

Table S1. Quantification of Sn(II) in TiSn5_C and TiSn5_H.

Sample	Peak B area	Sn(II) quantity (as percentage)
SnO_2	1.023	/
SnO	0.170	100%
TiSn5_C	0.582	51.7%
TiSn5_H	0.268	88.4%

References

1. Ryan, C.G.; Clayton, E.; Griffin, W.L.; Sie, S.H.; Cousens, D.R. SNIP, a statistics-sensitive background treatment for the quantitative analysis of PIXE spectra in geoscience applications. *Nucl. Inst. Methods Phys. Res. B* **1988**, *34*, 396–402.
